# Automated assessment of cardiac dynamics in aging and dilated cardiomyopathy Drosophila models using machine learning

**DOI:** 10.1038/s42003-024-06371-7

**Published:** 2024-06-07

**Authors:** Yash Melkani, Aniket Pant, Yiming Guo, Girish C. Melkani

**Affiliations:** 1 Department of Pathology, Division of Molecular and Cellular Pathology, Heersink School of Medicine, University of Alabama at Birmingham, Birmingham, AL USA; 2 Engineering Physics Department, College of Engineering, University of California, Berkeley, CA USA; 3 Department of Materials Science and Engineering, Georgia Institute of Technology, Atlanta, GA USA

**Keywords:** Machine learning, Data integration

## Abstract

The Drosophila model is pivotal in deciphering the pathophysiological underpinnings of various human ailments, notably aging and cardiovascular diseases. Cutting-edge imaging techniques and physiology yield vast high-resolution videos, demanding advanced analysis methods. Our platform leverages deep learning to segment optical microscopy images of Drosophila hearts, enabling the quantification of cardiac parameters in aging and dilated cardiomyopathy (DCM). Validation using experimental datasets confirms the efficacy of our aging model. We employ two innovative approaches deep-learning video classification and machine-learning based on cardiac parameters to predict fly aging, achieving accuracies of 83.3% (AUC 0.90) and 79.1%, (AUC 0.87) respectively. Moreover, we extend our deep-learning methodology to assess cardiac dysfunction associated with the knock-down of oxoglutarate dehydrogenase (OGDH), revealing its potential in studying DCM. This versatile approach promises accelerated cardiac assays for modeling various human diseases in Drosophila and holds promise for application in animal and human cardiac physiology under diverse conditions.

## Abstract (web summary)

A novel deep learning method is developed to analyse Drosophila hearts which is able to predict ageing and study cardiac dysfunction, offering potential for modeling human ailments in Drosophila

## Introduction

Cardiovascular disease (CVD) continues to be among the leading causes of death and disability in the United States and a major public health burden. Despite other risk factors, aging is one of the major risk factors for advancing CVD. The modern human population is increasingly adapting to lifestyles that further exacerbate CVD risks^1^. The *Drosophila melanogaster* (fruit flies) model system will allow us to comprehensively examine the potential cardiac benefit of a simple lifestyle modification and identify the relevant molecular and physiological changes. Gaining pathophysiological insights is essential for designing therapies. In vivo models such as *Drosophila* models and modern technologies including machine learning are powerful tools for understanding several aspects of human pathophysiology, such as aging and CVD. Despite some morphological and functional differences, *Drosophila* and human hearts’ conserved pathways appear to govern form and function^2–9^. Several genetic and non-genetic risks for heart diseases in humans also increase disease risks in *Drosophila* during aging. Moreover, mutant flies carrying genetic variants that are associated with a higher risk for CVD result in a similar outcome in the fly^3,4,6^. Furthermore, flies like humans living in industrial societies, consume some of their daily caloric intake at night and nutritional challenges that compromise cardiac function in humans have similar effects on flies^10–12^.

Progress in the high-speed recording of optical cardiac imagery has led to remarkable development in the application of *Drosophila*, zebrafish, and embryonic mouse hearts for cardiac modeling^13^. For example, current canonical methods using  the Semi-Automatic Optical Heart Analysis (SOHA) software offer methods for measuring relevant cardiac parameters during aging and under multiple disease conditions. However, the SOHA software requires manual selection of points of interest in diastole and systole phases to track changes in heart morphology. Dong et al. demonstrated the use of 3D convolutional architectures for the segmentation of *Drosophila* heart images studied via optical coherence microscopy (OCM) setups^14^. The authors reported robust segmentation (92% IoU) of OCM videos, which can calculate EDD, ESD, heart area, and heart rate. Similarly, Lee et al. demonstrate *Drosophila* heart-beat counting using segmentation of optical coherence tomography (OCT) recordings^15^. They demonstrate similar segmentation of OCM videos and apply the PCA procedure for heart morphology reconstruction. Work by Klassen et al. enabled in vivo imaging of *Drosophila* aging models with fluorescence imaging and conventional image-processing techniques for high-resolution, unanesthetized beating patterns^16^. Using a conventional computer vision approach, the authors provide methods for automatic segmentation and parameter (chamber diameter, fractional shortening, systolic interval, cardiac output, and heart wall velocity) calculations. We find much discussion of successful automatic analysis techniques for optical coherence microscopy but a lack of literature surrounding similar techniques in standard high-resolution optical microscopy setups, likely due to added complexity in visualized heart morphology.

Through literature review, we find that a limited amount of cardiac physiological data has been analyzed using machine learning from morphological data collected with OCT or OCM techniques, with quantification of all relevant cardiac parameters. Moreover, heart analyses using machine learning techniques in the literature have not tested rigorous *Drosophila* aging or disease models. Furthermore, there is a lack of automated methods for the analysis of high-speed *Drosophila* cardiac optical recordings in general optical microscopy setups. Modern video volumes outgrow the use of manual analysis techniques, necessitating automatic analysis methods. Our method differs from others in that it allows for automatic analysis of optical cardiac recordings at high spatial and temporal resolutions. Furthermore, we provide all cardiac statistics via a deep learning-assisted pipeline. We attempt a solution with a well-known deep learning-assisted medical image segmentation architecture, the UNet. Recent developments in machine learning have enabled state-of-the-art dense image segmentation platforms, such as the development of fully convolutional networks (FCNs)^17^, SegNets^18^, and UNets^19–22^. Historically, UNet architectures have been applied for cell nuclei, spleen, and liver segmentation^23,24^, COVID-19 prognosis^25^, and more. Human heart analysis has been a central focus in medical segmentation efforts^26–28^. In 2020, Ouyang et al.^26^ demonstrated the use of convolutional architectures for segmentation and beat-level analysis of human echocardiograms. They demonstrate real-time prediction of ejection fraction with comparable or less variance than that of human predictions. In this work, we draw inspiration from similar applications of medical image segmentation and apply UNet architectures to segmentation and beat-level analysis of *Drosophila* cardiac recordings.

To augment the human analysis of *Drosophila* cardiac recordings, we apply techniques in medical segmentation for the analysis of high-speed optical recordings. We calculate multiple diagnostic cardiac parameters for assessing cardiac function from videos alone. A 2D attention-based UNet architecture is applied for frame-level dense segmentation of cardiac recordings. Using only the generated annotations, we calculate beat-level cardiac parameters and beating patterns. Cardiac parameters are calculated on a per-beat resolution. We used $n=54$ hearts for model training and validated an experimental aging model with $n=177$ hearts. We investigate the use of both Drosophila cardiac statistics and cardiac videos for fly age prediction as an example downstream task. We determine that conventional machine learning and deep learning methods can be applied to predicting fly age, suggesting that morphological features evident in video recording can be an effective predictor of an aging phenotype.

Moreover, we have expanded the scope of our innovative deep-learning methodology beyond aging models to quantify cardiac physiological dysfunction associated with oxoglutarate dehydrogenase (OGDH), linked to dilated cardiomyopathy (DCM). OGDH is a pivotal enzyme in the TCA cycle, and previous studies have linked OGDH or oxoglutarate dehydrogenase-like (OGDHL) with energy metabolism, neurodegenerative disease, aging, cancer, and skeletal muscle dysfunction^29–33^. Moreover, altered expression of OGDHL has been reported in the heart samples obtained from DCM patients, thus indicating a potential link between OGDHL and DCM^34^. However, we have developed an innovative *Drosophila* model that links OGDH to DCM, using SOHA and machine learning approach. Previously, we have developed Drosophila DCM models upon expression of mutant Htt-PolyQ as well as with laminopathy mutation^35,36^. Thus, this development highlights the broader applicability of our deep-learning approach, extending its significance from aging models to the assessment of pathological conditions like DCM, holding substantial translational potential.

This deep-learning approach to *Drosophila* cardiac analysis facilitates robust analysis of *Drosophila* cardiac physiology and enables access to future downstream analysis techniques. In addition to aging models, we demonstrate the translational potential of the presented deep-learning methodology by quantifying compromised cardiac performance and other physiological dysfunction associated with the knock-down of OGDH, a novel gene linked with DCM. The presented approaches can expedite future cardiac assays for modeling additional human diseases in *Drosophila* and can be extended to animal models and human cardiac assays under development, pathological, and aging conditions.

## Results

### Overview of the presented machine learning pipeline

**Fig. 1:**
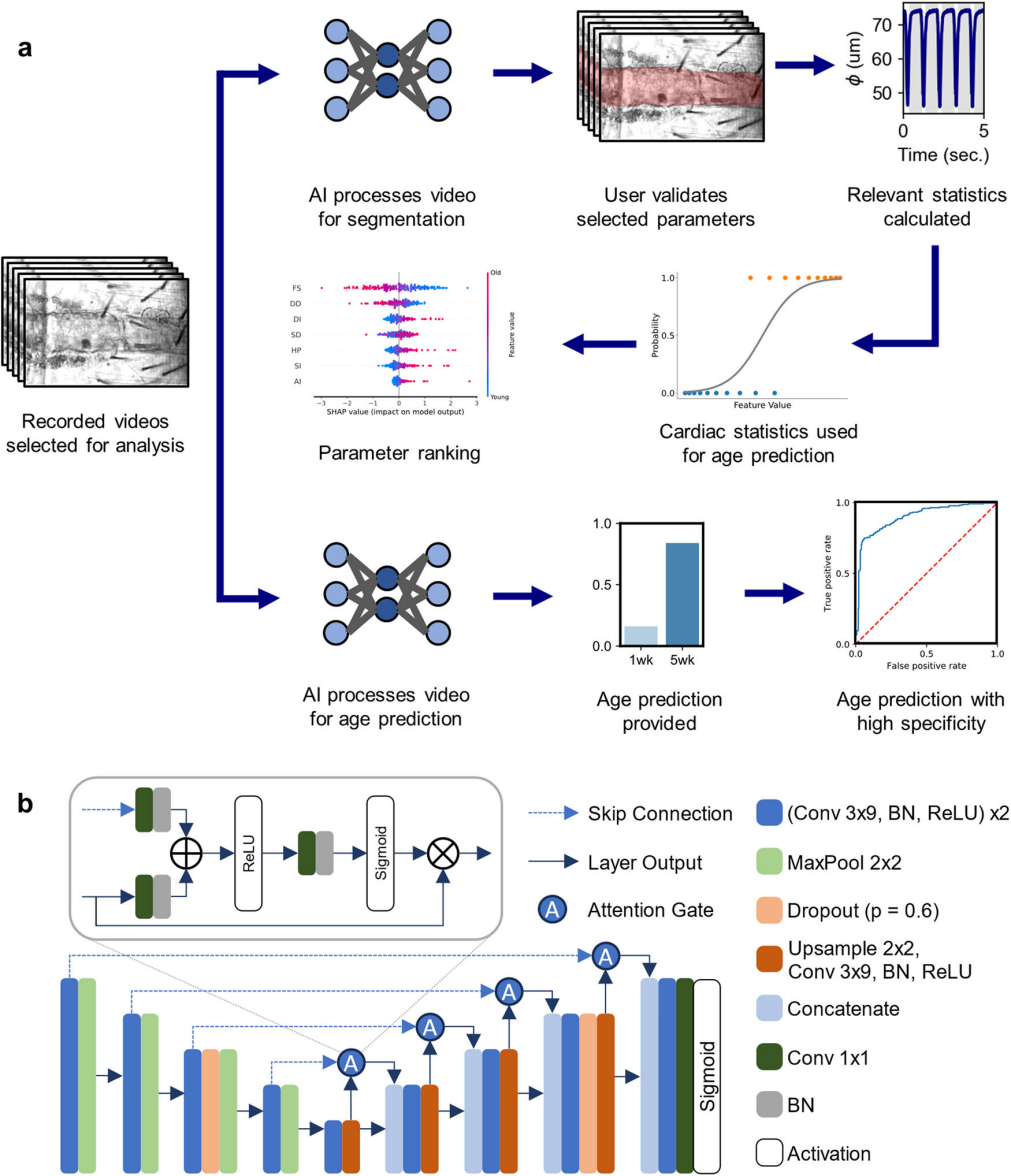
Proposed deep learning pipeline. **a** One pipeline enables frame-level segmentation of heart walls. For this task, users upload videos for analysis, perform segmentation using a trained neural network, select validated parameters, and extract beat-level parameters. This feeds into the middle pipeline which enables machine learning-based age-prediction of hearts via calculated cardiac statistics, further enabling transparent modeling of physiological parameters. Lastly, the bottom-most pipeline details an age-prediction task using a neural network, thus providing an alternate method for age-prediction. **b** Schematic of the presented segmentation model. We use an attention-UNet with rectangular convolution kernels. A diagram of the attention gates is also shown.

Analysis of *Drosophila* high-speed cardiac recordings requires high-throughput and accurate measurement techniques. Current methods involve tedious analyses with large amounts of personnel hours, necessitating automatic next-generation methods. In this work, we propose three machine learning-enabled pipelines that are well-suited for rapid analysis of *Drosophila* cardiac recordings. As depicted in Fig. 1a, we establish two unique tasks: (1) heart wall detection via semantic segmentation and (2) age prediction. In the presented segmentation pipeline (Fig. 1a), a user begins by uploading high-speed cardiac recordings to a central workstation via a file-transfer protocol. After this, the user selects videos for segmentation and analysis and provides them to our segmentation model, which is made accessible via a PyTorch interface^37^. Then, using our segmentation model, heart wall regions are tagged on a frame-by-frame basis, allowing us to easily calculate the average heart diameter and area per frame. A diagram of our segmentation model is shown in Fig. 1b. After results have been generated per frame, users may vary network parameters such as selecting a region-of-interest in diameter calculations and a model confidence threshold. Supplementary Fig. 1 Supplementary Video 1 (ZIPP File) demonstrates a video-format output of our network results for a given heart, for five seconds. Once a satisfactory segmentation is acquired, users may export time-resolved morphological data. Finally, using the exported morphological data, we make available relevant cardiac statistics for assessing function and rhythmicity. Example Python notebooks are provided for segmenting and analyzing heart videos with easy-to-use, interactive user interfaces.

Two separate pipelines are presented for age prediction. One such pipeline involves using segmentation-calculated cardiac statistics for age prediction. Age prediction via cardiac statistics is powered by a logistic classification model. Such a logistic classification approach enables transparent reporting of model interpretation via black-box methods such as the calculation of SHAP scores. Furthermore, we present a fully deep learning-based pipeline for age classification. Through the use of raw video frames, we determine that convolutional neural networks can predict fly age with high accuracy and specificity. Additional detail for both methods is provided in the Method section.

### Representative output from our deep learning pipeline

**Fig. 2:**
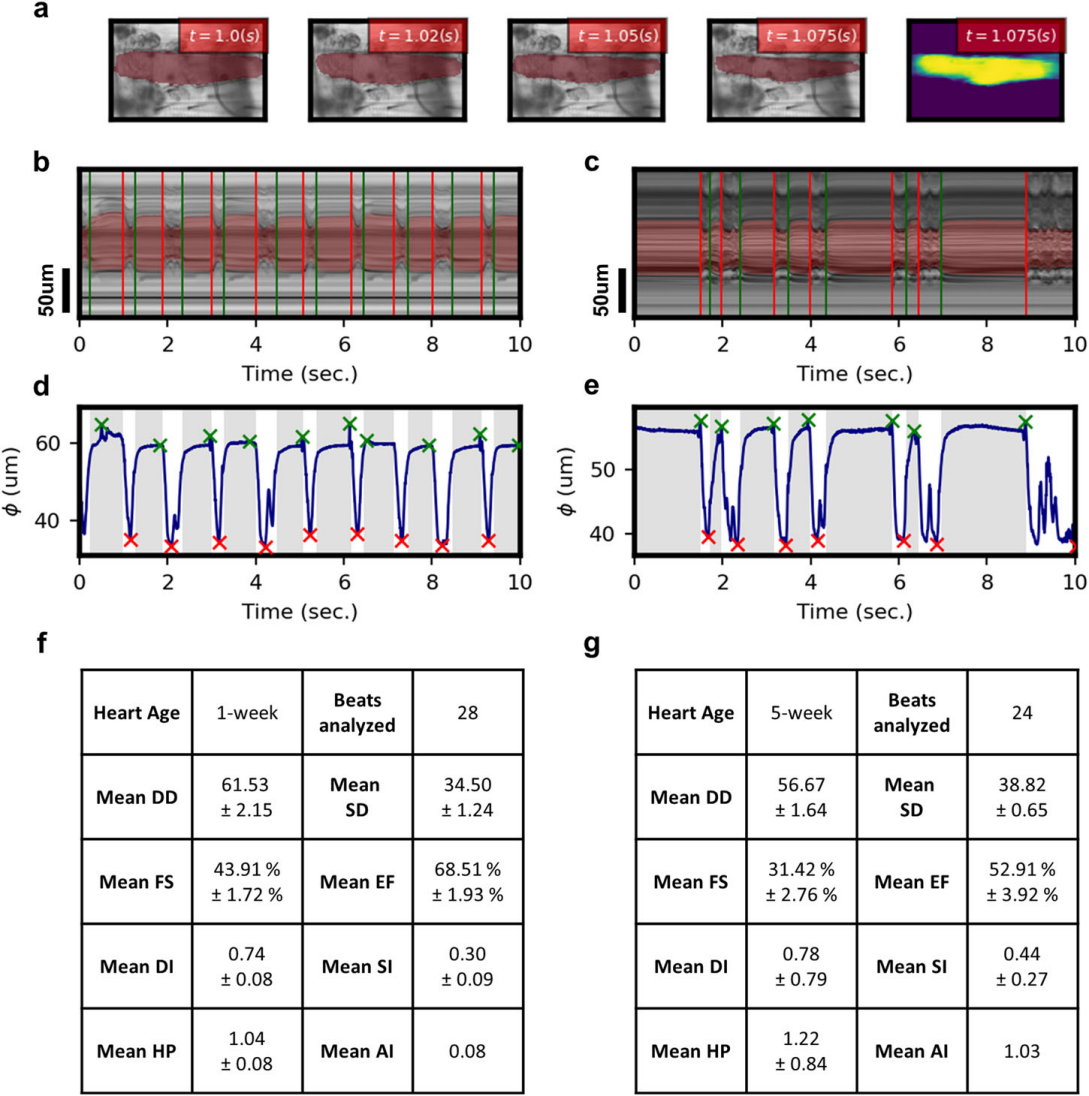
Representative output of the neural network. **a** Select frame extracted from cardiac video, with the overlaid mask from a neural network denoting the heart wall. **b**, **c** Using annotated frames, an annotated mechanical mode (M-Mode) image is generated. **d**, **e** Detected heart masks are used to calculate average stationary diameter on a per frame resolution. **b**, **d**, **f** represent generated M-Mode, time-series beating pattern, and calculated cardiac parameters for a representative 1w male heart. **c**, **e**, **g** represents generated M-Mode, time-series beating pattern, and calculated cardiac parameters for a representative 5w male heart. Green (start of DI) and red (end of DI) lines are annotated in **b, c**, and gray periods in **d**, **e**. Times of max contraction (red) and max relaxation (green) are annotated by x indicators in **d**, **e**.

**Fig. 3:**
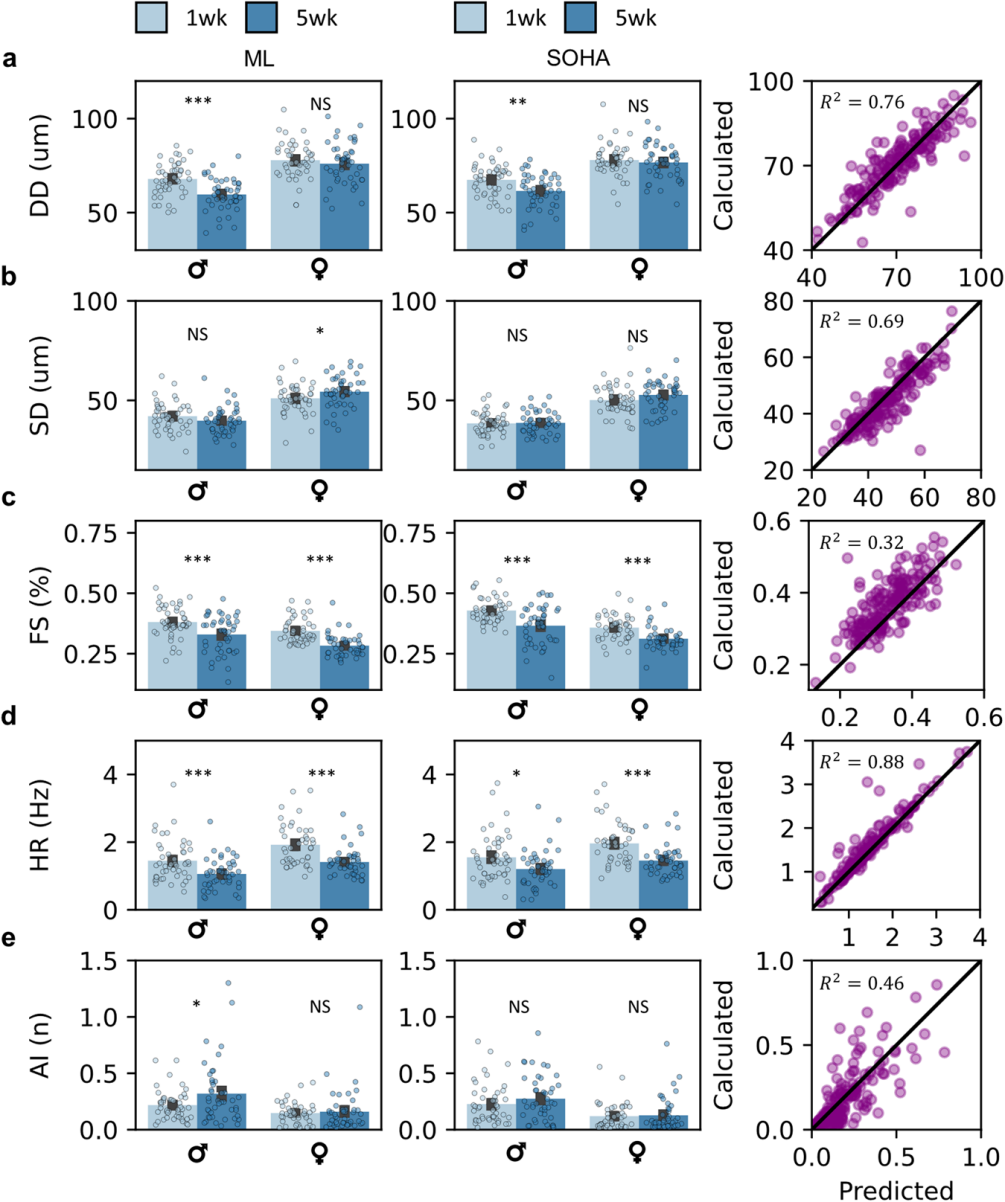
Deep learning recovers aging phenotypes in the Drosophila cardiac model. **a** Diastolic diameter, in micron, is calculated by our model (left), SOHA (middle), and agreement between two datasets (right). **b** Systolic diameter, in microns, calculated by our model (left), SOHA (middle), and agreement between two datasets (right). **c** Radial contractility (fractional shortening), in percentage, calculated by our model (left), SOHA (middle), and agreement between two datasets (right). **d** Heart rate, in Hertz, is calculated by our model (left), SOHA (middle), and agreement between two datasets (right). **e** Beating dysrhythmia (arrhythmia index) calculated by our model (left), SOHA (middle), and agreement between two datasets (right). All error bars report ± SEM. Age-dependent statistics compared with one-way ANOVA with two-sided unpaired t-test. Statistics are calculated via the use of a restricted ROI, selected by a trained user.

**Fig. 4:**
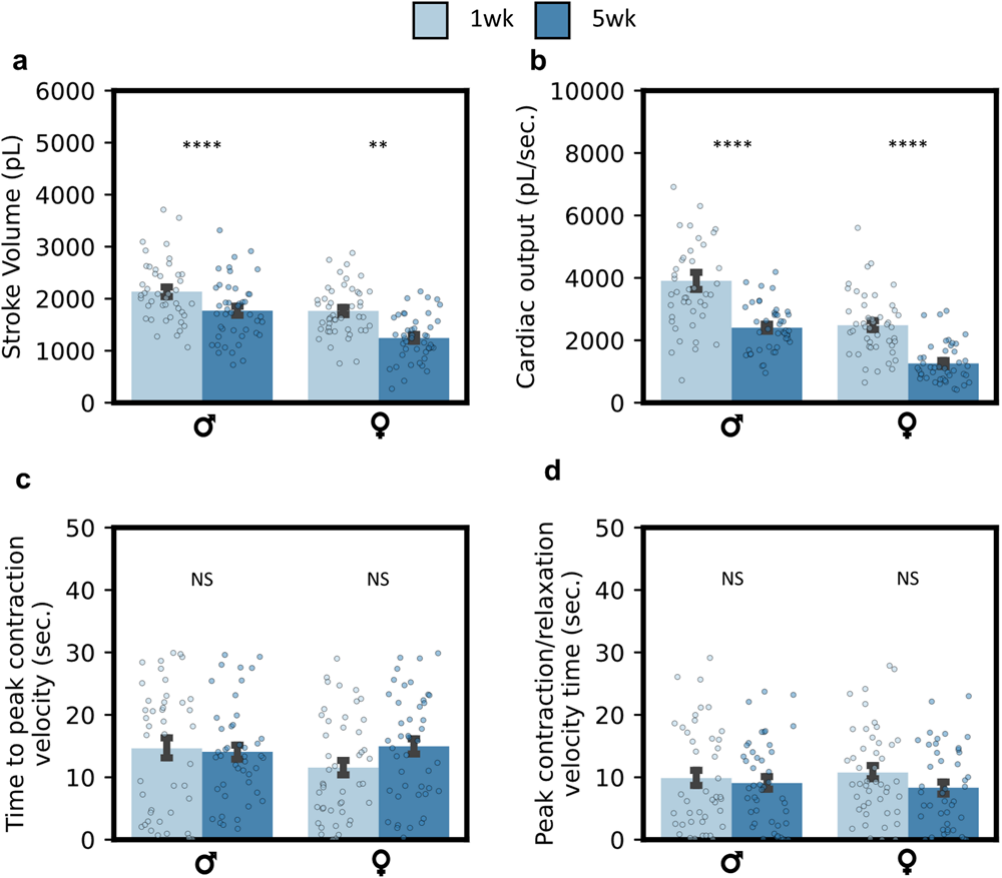
Contractile dynamics detected by deep learning. **a** Stroke volume for male and female aging groups is depicted, in picolitres. A significant reduction in stroke volume is exhibited with aging in both genders. **b** Cardiac output was visualized via integration of per-beat stroke volumes and normalized by beat times, measured in pL s-1. Aging depicts a strong reduction in cardiac output. **c** Time to peak contraction (negative) heart-wall velocity visualized for aging. **d** The time between peak contraction (negative) and relaxation (positive) velocities visualized for aging. Both **c** and **d** reflect no significant change with aging. All error bars report ± SEM. Age-dependent statistics compared with one-way ANOVA with two-sided unpaired t-test t.

Figure 2 demonstrates a representative output from our deep learning pipeline. This includes frame-by-frame heart wall output, visualized beating patterns, time-resolved morphological data, and calculated cardiac statistics on a representative level. Heart morphology is captured on a per-frame basis, denoted by overlaid red layers (Fig. 2a), as indicated in the methods section. Qualitative inspection suggests excellent agreement between detected heart regions and ground truth through all stages of the cardiac cycle. Figure 2b, d, and f present representative model outputs for a 1-week male heart. Alternatively, Fig. 2c, e, g present representative model outputs for a 5-week male heart. We trace the annotation over time at a single vertical slice, creating a mechanical mode (M-Mode) image, demonstrated in Fig. 2b, c. Again, one may note excellent agreement between annotated and ground truth M-Mode, indicating excellent capture of the heart wall dynamics. Periods of diastolic and systolic intervals are automatically labeled with red and green vertical lines. Figure 2d, e depicts time-resolved beating patterns constructed via measurement of heart diameter through segmented heart walls. The average diameter is calculated by measuring each vertical pixel-slice distance in our frame-by-frame heart walls. This is further discussed in the Methods section. The time-resolved beating patterns demonstrate differences in contractility and arrhythmia, making neural-network-captured aging phenotypes clear on a representative basis. These are further quantified in tabulated cardiac statistics for a single heart $(n=1)$, available in Fig. 2f, g. These aging phenotypes are presented for a larger-scale study in Figs. 3 and 4.

### Experimental validation of the Drosophila aging model

A study investigating cardiac aging phenotypes was performed on a cohort population. We demonstrate that our model can capture aging phenotypes presented in existing literature with a fraction of the effort required from canonical analysis techniques (SOHA). We calculate a full suite of cardiac statistics including diastolic diameter (Fig. 3a), systolic diameter (Fig. 3b), fractional shortening (Fig. 3c), heart rate (Fig. 3d), and arrhythmia index (Fig. 3e). Additionally, we provide select temporal statistics including heart period, diastolic intervals, and systolic intervals in the SI (Supplementary Fig. 2). Figure 4 presents model-detected contractile dynamics, including aging phenotypes in spatial and temporal modes. Supplementary Fig. 3 depicts heart fractional shortening and heart period, as a function of gender and age at the beat level.

### Deep learning recovers aging phenotypes including contractile dynamics in a Drosophila cardiac model

We find that expected directional trends in cardiac function and arrhythmia are captured successfully across age groups and genders (Fig. 3). Spatial (cardiac morphology) associated statistics are discussed. We demonstrated a statistically significant decrease in diastolic diameter in male aging groups and no change in diastolic diameter across female aging groups, with agreement between experimental machine-learning (ML) and SOHA analyses. Systolic diameter demonstrates little variance across age groups with the agreement between ML SOHA groups, except for a weak statistically significant difference in the ML female aging group $(p=0.049)$. Furthermore, we observe a strong statistically significant decrease in cardiac function (fractional shortening) across both male and female aging $(p < 0.001)$. ${R}^{2}$ measurements between ML and SOHA data are high for diastolic and systolic diameter measurements, at ${R}^{2}=0.76$ and ${R}^{2}=0.69$, respectively. However, ${R}^{2}$ is low for fractional shortening measurements ${R}^{2}=0.32$. We demonstrate a strong reduction in cardiac function with age across both male and female groups in ML and SOHA studies, as expected. This agrees with morphological parameters quantified in Fig. 4, with a statistically significant reduction in stroke volume (Fig. 4a) and cardiac output (Fig. 4b). Methods for calculating all discussed parameters are made available in the Methods section.

Next, we discuss temporal-associated cardiac statistics. We find that our model can accurately capture changes in heart rate, with expected significant differences across age groups in both ML and SOHA data points. A similar agreement is presented in studied heart period, diastolic, and systolic interval data across ML and SOHA groups. The model captures an accurate evolution of cardiac arrhythmia in female aging data in both ML and SOHA groups but exhibits an unexpectedly significant difference in male aging groups. ${R}^{2}$ measurements between ML and SOHA data are high for temporal statistics including heart rate, heart period, diastolic interval, and systolic intervals, at ${R}^{2}=0.88,{R}^{2}=0.91,{R}^{2}=0.89,$ and ${R}^{2}=0.63,$ respectively. However, ${R}^{2}$ is low for arrhythmia index measurements at ${R}^{2}=0.46$. We observe a small increase in cardiac arrythmia with aging in both ML and SOHA groups. While heart period and derivative temporal parameters demonstrate statistically significant shifts with aging, changes in rhythmicity and contractile latency are not exhibited with aging (Figs. 3 and 4). Latency is quantified in Fig. 4c, d, concluding that no significant difference in latency to peak contraction and latency between peak contraction to peak relaxation is expressed in our aging model across aging in gender-separated data. This signals agreement with previously presented results in measured arrhythmia shifts with aging.

We also demonstrate our ability to capture beat-level contractile and temporal dynamics in beating patterns. In both male and female groups, we see a large flattening across 1-week and 5-week fractional shortening distributions, indicating a large increase in contractility variance (Supplementary Fig. 3a, b). Furthermore, a general decrease in contractility with aging is depicted on a beat level, as expected from cohort-level data. The reverse trend is true in heart period distribution, with flattening across both male and female aging groups but an increase, on average, in beat length (Supplementary Fig. 3c, d). Furthermore, we employ beat-level dynamics to detect brachy- and tachy-cardiac arrhythmia. Supplementary Fig. 4 quantifies the prevalence and significant shifts in exhibited tachy- and brachy-cardiac arrhythmia across our aging model. We find a significant increase in average SI length during tachycardiac events for male specimens with aging (Supplementary Fig. 4). Additionally, we observe a significant increase in average DI length during brachy-cardiac events for male specimens through aging. We note an exceptionally small $n$-value at *n* = 2 for bradycardia events in young female hearts. We visualize cohort-level and beat-level data for SI and DI distributions (Supplementary Fig. 4c). Beat-level data suggests a widening distribution in both SI and DI for aging male and female specimens.

### Prediction of aging in Drosophila data

**Fig. 5:**
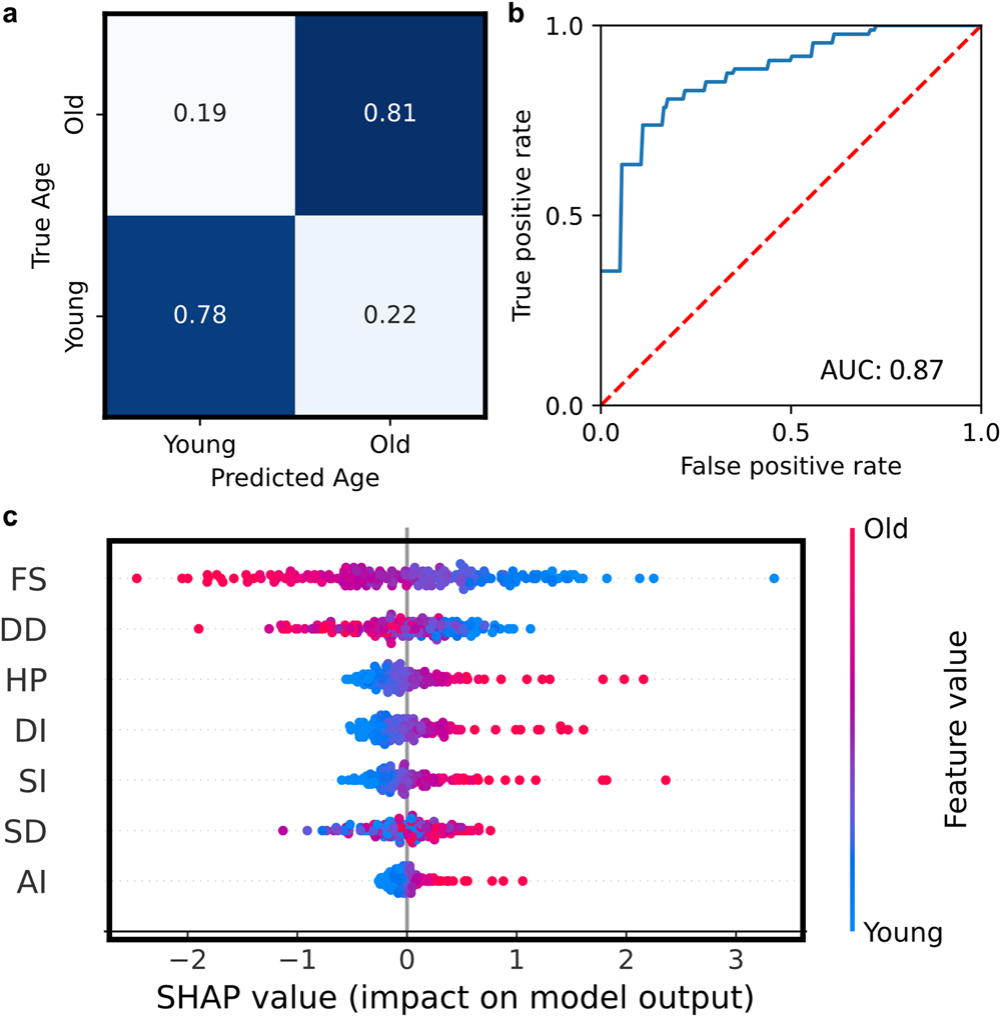
Machine learning classification of aging data. Logistic classification models enable accurate prediction of fly age from model-calculated cardiac statistics. **a** Heatmap of test-dataset predictions, indicating high agreement. **b** Average ROC-curve of all folds from the presented model, reporting a mean AUROC of 0.87. **c** Physiological feature relevancies for predicting fly age, as determined by the SHAP study. SHAP study suggests a large dependence on fractional shortening (FS) in aging.

**Fig. 6:**
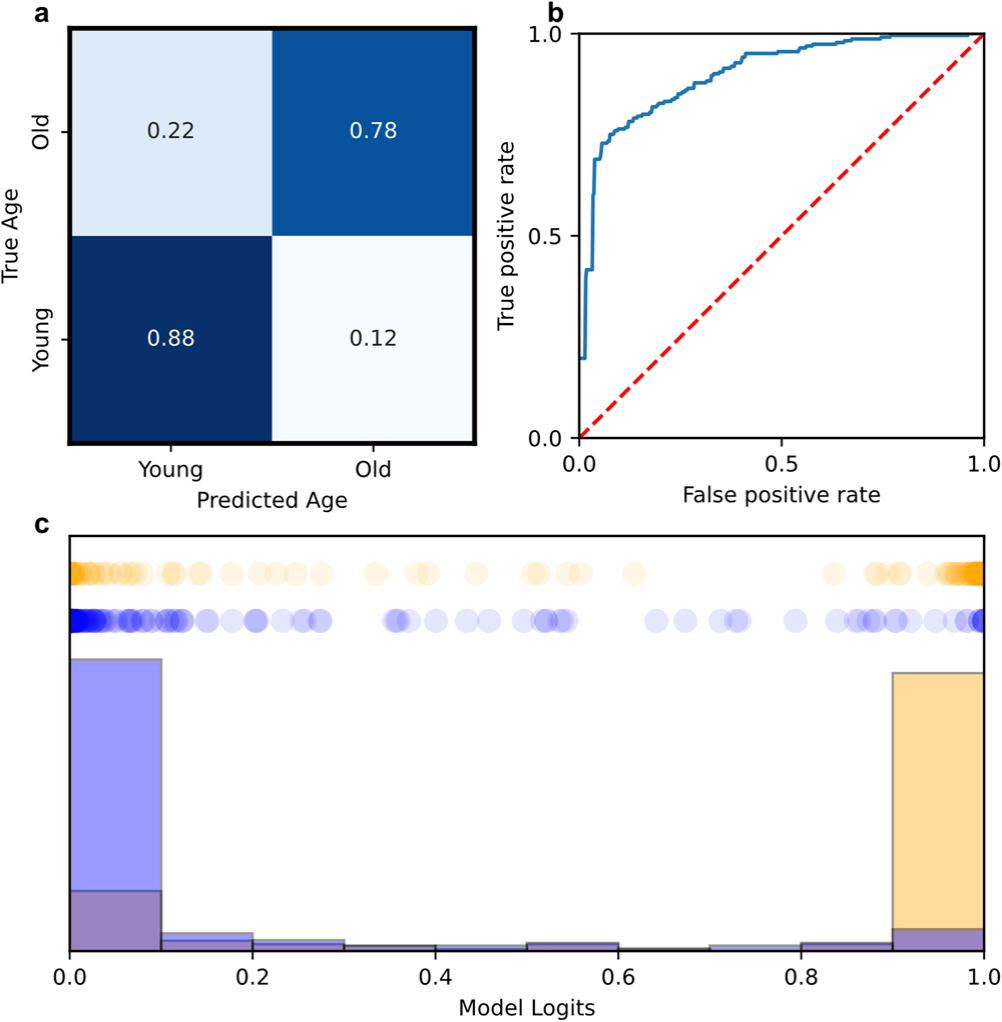
Deep learning classification of aging videos (n = 497). Deep learning models enable accurate prediction of fly age from only video data. **a** Heatmap of test-dataset predictions, indicating high agreement. **b** Average ROC curve of all folds from the presented model, reporting a mean test accuracy of 83.3% and mean AUROC of 0.900. **c** Model class log-likelihood distribution for investigated test predictions.

We investigate the use of machine learning approaches for age classification in *Drosophila* datasets (Figs. 5 and 6). We apply logistic regression for age classification (Fig. 5). We note high accuracy in experimental classification, with an accuracy of 79.1% and AUROC of 0.87 (Fig. 5a, b). We observe the highest inaccuracies to be found in young hearts falsely classified as old. The presented results suggest that cardiac statistics are accurate predictors of aging phenotypes. We demonstrate the use of SHAP values for assessing drivers of aging function, enabling interpretable machine learning (Fig. 5c). Descriptors are sorted in predictive importance, from top to bottom. Thus, we note that SHAP methods determine fractional shortening (FS) as a main predictor of fly age, followed by diastolic diameter (DD).

Using a deep convolutional neural network, we train a video classifier for assessing fly age from cardiac video without knowledge of cardiac parameters. Results of this technique are presented in Fig. 6. The constructed model architecture along with data preparation are discussed in the Methods section. Using a k-fold cross-validation procedure, we perform experimental validation on $n=497$ cardiac samples, with group breakdowns of $n=118$ for 1wm, $n=156$ for 1wf, $n=121$ for 5wm, and $n=98$ for 5wf. We observe an average AUROC score of 0.90 (Fig. 6b) and an average accuracy of 83.3%, demonstrating excellent performance, further depicted in the confusion matrix (Fig. 6a). Qualitatively, we note excellent separation of age likelihood as determined by our model, with a high separation between young and old likelihoods (Fig. 6c). The presented results suggest a neural architecture can recover fly age from cardiac video based on spatial data from select frames. We note that the highest number of inaccuracies found in old hearts falsely predicted as young (22% of samples). Overall, we determine that deep learning classification models can model aging directly on raw cardiac video with high accuracy, suggesting such models may possess capabilities for capturing morphological or rhythmic features in video inputs. Furthermore, we determined that both cardiac parameters and videos can be applied for highly accurate aging prediction in *Drosophila* models.

### Deep learning approach for dilated cardiomyopathy contractile dynamics and compromised cardiac performance in a Drosophila cardiac model

**Fig. 7:**
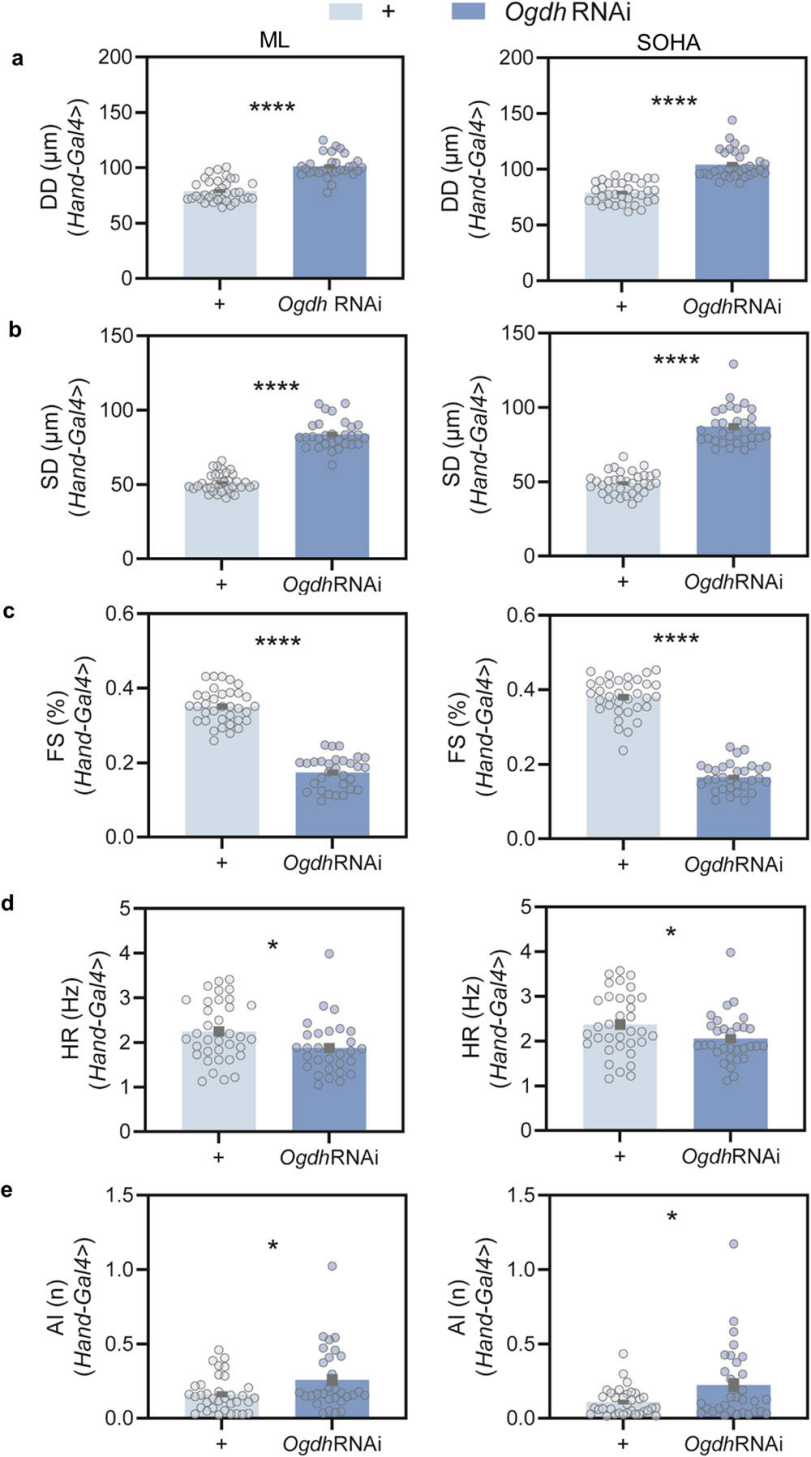
Cardiac-specific knock-down of *Odgh* leads to cardiac dilation and compromised cardiac performance. Histograms of cardiac physiological parameters 3-weeks control (*Hand/+)* and *Hand>Ogdh RNAi* has compromised **a** diastolic diameter (DD), **b** systolic diameter (SD), **c** % fractional shortening (FS), **d** heart rate (HR), and **e** arrhythmia Index (AI) analyzed using machine learning (ML) left, and SOHA right panels. *n* = 35 (*Hand/+)* and 30 *Hand>Ogdh RNAi for* ML; and *n* = 34, (*Hand/+)* and 31 *Hand>Ogdh RNAi* for SOHA were represented as mean ± SEM. Statistical analysis of each cardiac parameter between control and *Ogdh* knock-down was carried out using one-way ANOVA with two-sided unpaired t-test.

In addition to the aging model, we have tested the broader applicability of our deep-learning approach by quantifying cardiac physiological dysfunction for the assessment of pathological conditions like DCM. As shown in Fig. 7a–c, cardiac-specific *knock-down (KD)* of oxoglutarate dehydrogenase (OGDH), a pivotal enzyme in the TCA cycle, in 3-week-old female flies leads to severe cardiac dilations, shown with significantly enhanced diastolic diameters (DD) and systolic diameters (SD), which lead to a significant reduction with cardiac performance (%FS), compared to age-matched control. These are the features of dilated cardiomyopathy (DCM) as we and others have previously reported. As shown for the aging studies, our ML and SOHA data showed the same trends in modeling DCM (Fig. 7a–c). Moreover, other cardiac physiological parameters including heart rate (HR) were found to be reduced and arrhythmia index (AI) was found enhanced upon cardiac-specific *KD* of *Ogdh* in 3-week-old flies, compared to age-matched control (Fig. 7d, e). Furthermore, as shown in Supplementary Fig. 7a–c, cardiac-specific *KD* of *Ogdh* in 3-week-old flies, compared to age-matched control led to compromised cardiac physiological of other parameters including long diastolic and systolic intervals (DI and SI), and heart period (HP). Male flies also showed a similar trend in all the cardiac parameters. Thus, the development of the ML data analysis approach is useful for the assessment of DCM highlighting and extending its significance in ML beyond aging models. Furthermore, we choose to compare the distributions of our aging model predictions for the 3wk control group $(n=35)$ and the 3wk DCM group ($n=30$). As demonstrated in Supplementary Fig. 8, the two distributions are qualitative like each other, which suggests that despite significant differences in cardiac physiological parameters between control and DCM, the latter does not contain the markers of accelerated aging.

## Discussion

We present methods for the machine and deep-learning analysis of Drosophila optical cardiac videos. We find that deep segmentation models can accurately recover important contractile and temporal dynamics in *Drosophila* heart beating patterns. Our approach allows us to bypass the time-consuming human involvement required in existing canonical software such as SOHA^13^. Moreover, our automated machine-learning analysis will help erase human errors in marking heart edges under contraction and relaxation conditions. This is one of the vital steps for automated analysis as diastolic and systolic diameters as well as fractional shortening (cardiac performance) depend on accurately marking cardiac edges. Such cardiac edge marking is automated in Dong et al.^14^ for OCM and Klassen et al.^16^ for fluorescent microscopy but is yet to be automated in standard high-resolution optical microscopy platforms. Dong et al.^14^ provide utilities for heartbeat calculation. We provide a full suite of cardiac statistics that may be automatically calculated using our deep model. The presented code can readily provide calculated cardiac statistics including diastolic and systolic diameters/intervals, fractional shortening, ejection fraction, heart period/rate as well as quantifying heartbeat arrhythmicity. Our model may be applied to readily available consumer hardware. In our study, we employ the Tesla P100 but suggest that model inference can be carried out with lower-end, consumer graphics models. Our method may potentially aid researchers with a higher fidelity, reproducible, and more automatic approach to *Drosophila* cardiac modeling beyond the capabilities of human technicians.

We use a 2D deep segmentation model to generate heart-wall segmentations on a frame-by-frame basis. We find that the employed segmentation model can recover accurate heart-wall segmentations, allowing us to track frame-resolved morphology as well as relevant cardiac statistics on a beat-by-beat resolution. Using our deep learning method, we note fly aging expresses a significant reduction in cardiac function (contractility) and an increase in cardiac dysrhythmia. Similarly, our model detects significant changes in aged heart rate and heart period, as well as underlying parameters including diastolic and systolic intervals. Furthermore, such annotations open opportunities in precise time-resolved study of *Drosophila* cardiac morphologies in optical micrography assays (Fig. 2). Such analysis is not possible using canonical analysis software. For example, deep learning-assisted modeling of cardiac diseases in *Drosophila* cardiac morphologies may reveal unique physiological information^36,37^.

To our knowledge, this innovative platform for deep learning-assisted segmentation is the first of its kind to be applied to standard high-resolution, high-speed optical microscopy of *Drosophila* hearts while also quantifying all relevant parameters (Figs. 3 and 4, and Supplementary Fig. 2). Cited works such as Ouyang et al.^26^ reports a limited amount of cardiac parameters and provides ejection fraction as a model-derived value on human echocardiography data. Ouyang et al. studied failing hearts as a contrasting model to demonstrate differences in beat-level ejection fraction but did not include age dependence. Similarly, Lee et al.^15^ report segmentation on *Drosophila* OCM video, with fewer provided cardiac parameters including heart rate, ESD, EDD, and FS, with some discussion on aging phenotypes. We further enable understanding of contractile dynamics via our beat-level capabilities (Figs. 3, 4, Supplementary Fig. 3). Through per-frame analysis, we quantify contractility through measurement of morphological parameters quantifying stroke performance and latency (Fig. 4a, b). We note a significant reduction in spatial beating modes (stroke volume and cardiac output) with aging, but little to no dependence on aging for temporal beating character (time to peak contraction velocity and time between peak relaxation and contraction velocities) (Fig. 4c, d). However, we do note significant shifts with aging in beat lengths, indicated in the modeling of HP and derivative parameters. Beat-level investigation elucidates per-beat information regarding cardiac arrythmia including tachycardia and brachy-cardiac arrythmia (Supplementary Fig. 4). We observe a significant, large increase in DI length during brachy-cardiac events in 5wk male flies.

We demonstrate capabilities of predicting fly age using experimentally calculated cardiac statistics with excellent agreement (Fig. 5). We also use a 2D video classification model to predict fly ages between 1-week and 5-week groups (Fig. 6). The ability to classify age via cardiac statistics suggests that experimentally calculated cardiac statistics are physiologically salient and model age dependence. Additionally, the ability to predict age with raw videos suggests that deep learning models can capture morphological and rhythmic patterns in *Drosophila* cardiac video data. This has important implications for detecting phenotypes mimicking or delaying aging of *Drosophila* hearts. To our knowledge, our innovative work determined that deep neural networks could capture cardiac physiological features of the heart that induce aging and cardiomyopathy in cardiac videos collected with optical microscopy. In the future, such classification could be extended to classify healthy and compromised hearts linked with other cardiovascular disease models and may include further quantification through methods such as GradCAM^38^.

In addition to the aging model, we have successfully implemented a deep-learning methodology that quantified compromised cardiac function in a DCM model, associated with the cardiac-specific knock-down of *Ogdh*. OGDH or oxoglutarate dehydrogenase is an enzyme in the TCA cycle, which is essential for ATP production in the cardiac myocytes. Disruption of OGDH compromisees energy production, a critical process for cardiac contractility. Further, altered expression of oxoglutarate dehydrogenase-like (OGDHL) has been reported in the heart samples obtained from DCM patients, thus indicating a potential link with DCM^34^. Mutations in OGDH have been linked with metabolic disease and OGDH deficiencies have been linked with neurodegenerative disease in humans. The role of OGDH or OGDHL has therefore been linked with energy metabolism, cardiac, neurodegenerative disease, aging, cancer, and skeletal muscle dysfunction^29–33^. However, no model exists for OGDH involvement with DCM. We have developed an innovative *Drosophila* model that links OGDH to DCM, using SOHA and machine learning approaches (Fig. 7 and Supplementary Fig. 7). Cardiac-specific knockdown of Odgh resulted in DCM, consistent with our previous findings in models of human dilated cardiomyopathy caused by mutant Htt-PolyQ or laminopathy mutations^35,36^. More studies will be required to further establish the mechanistic basis of DCM-linked OGDH, however, since OGDH is located in the mitochondria the energy requirement of cardiac myocytes might have compromised, resulting in significantly reduced cardiac performance^39^. Future studies will be relevant to improve cardiac energy metabolism, which can be a potential therapeutic target to suppress DCM.

Current limitations include validation of parameters including calculation region of interest and suitable confidence thresholds. In the future, we hope to overcome this limitation. We report preliminary results in Supplementary Figs. 5 and 6 using our trained segmentation approach without specifying a region of interest for morphological calculation (referred to as “No ROI”). Furthermore, we homogenously employ a pre-selected threshold, enabling a user to analyze samples with no human input or supervision. The presented results demonstrate excellent agreement in temporal statistics, including high accuracy of all the parameters in DCM models. However, in the aging model, heart rate/period, diastolic and systolic intervals demonstrate excellent agreement in temporal statistics, but disagreement in the arrhythmia index. Spatial statistics, however, demonstrate strong disagreement in systolic diameter, driving further disagreement in fractional shortening. This could be due to beat-to-beat variability in each parameter and is likely exacerbated by the limited size of our labeled dataset. As shown in Supplementary Table 1, our labeled dataset only consists of 54 cardiac recordings from a handful of genetic conditions. We heavily rely on dataset balancing and image augmentations during training. More training data from a more diverse set of genetic and environmental contexts would lead to stronger spatial performance in the case of completely hands-free usage. Larger datasets, however, would also require further developments in domain adaptability, self-supervised training objectives, and sophisticated model architectures. We believe that these implementations would improve predicted cardiac parameter accuracy across the board.

It is known that several factors including genetic and environmental conditions can affect aging. This is particularly important as it is also known that certain genetic contexts can provide insight as to what physiological factors are relevant to predicting fly age. For instance, as we demonstrate, the distributions of aging model predictions for 3-week-old flies with DCM (*Ogdh* knockdown) and age-matched wild-type flies are qualitatively very similar. This suggests that the DCM model is a very weak factor in age prediction. However, except for the genetic factor (*Ogdh* knockdown), we did not evaluate the impact of environmental factors in influencing aging, which may be considered one of the limitations of our age prediction model. Overall, with our age-prediction model in its current form, it is possible to decipher the qualitative relevance of different physiological factors such as genetic conditions. In the future, we hope to expand the capabilities of our age prediction model to predict any fly age between 1wk and 7wk. Predicting age as a regression or a multi-class classification task allows users to statistically quantify the effect of different environmental and/or genetic factors on aging simply by comparing the distributions of model-predicted age of each cohort.

The *Drosophila* model has proven tremendously powerful for understanding the pathophysiological bases of several human cardiovascular diseases. Moreover, the substantial volumes of *Drosophila* cardiac data collected in the lab necessitate advanced methods for automated analysis of cardiac physiologies and morphologies on a beat-by-beat basis. In summary, we evaluate the use of deep segmentation models for high-fidelity analysis of cardiac physiologies in high-speed *Drosophila* cardiac optical recordings. We demonstrate that the presented deep segmentation model can be applied for accurately expressing known *Drosophila* phenotypes in aging across male and female 1-week and 5-week groups. Furthermore, we demonstrate that developed deep video classification methods can be successfully applied to Drosophila fly age classification using only video clips with exceptional accuracy. Importantly, the significance of this deep-learning approach extends beyond just the study of aging models, it holds considerable promise in the evaluation of pathological conditions, notably DCM. This development highlights the broader applicability of our deep-learning approach, extending its significance from aging models to the assessment of pathological conditions like DCM, holding substantial translational potential.

We hope these models can be applied in the future to expedite laboratory analysis and power next-generation *Drosophila* cardiological model analysis. Future applications of the discussed techniques include enabling deep learning-assisted studies of cardiac mutation models, other small animal models (e.g., commonly studied zebrafish and mice models), and parameter calculation in human heart models. Furthermore, quantification of measured uncertainty techniques may be applied to qualify certainty of heart analyses.

## Methods

### Drosophila stocks and conditions

All the experimental flies were housed within environmentally controlled incubators, maintaining a constant temperature of 25 °C, humidity at 50%, with and a strictly adhered to the 12-h light-dark cycle as previously reported^6,11,33,35,36^. Briefly, for the aging study wild-type *Canton-S* (Bloomington Drosophila Stock Center) flies were raised with a standard regular diet: agar 11 g/L, active dry yeast 30 g/L, yellow cornmeal 55 g/L, molasses 72 mL/L, 10% nipagen 8 mL/L, propionic acid 6 mL/L. Notably, adult flies were harvested immediately upon eclosion, meticulously sorted by gender, and subsequently aged to the appropriate age. Throughout the study, a standardized protocol was employed, housing 25 flies per vial as previously reported.

To assess the heart-specific function of *OGDH*, the GAL4-UAS system^40,41^ was used to drive the knockdown *Ogdh* specifically in the heart using the *Hand-Gal4* driver as previously reported^6,35,36^. *Hand-Gal4* was obtained from Dr. Olson’s lab. Transgenic stocks were obtained from Bloomington Drosophila Stock Center (BDSC) and Vienna Drosophila Resource Center (VDRC): UAS-*Ogdh*-RNAi (BDSC: 33686; VDRC:50393)^33^. Adult flies possessing UAS-*Ogdh*-RNAi were crossed to *Hand-Gal4* and incubated at 25°C throughout development. Adult male and female F1 progeny were separated according to sex and allowed to age, with a new food source supplied every three days before assays of cardiac function. Age-matched adults from *w*^*1118*^ (wild-type), were crossed with the *Hand*-*Gal4* driver as control. Female flies were screened at 3 weeks of age more than 30 each. Like the aging model, all flies were kept at 25 °C, 50% humidity in a 12-h light-dark cycle.

### High-speed cardiac recording

Physiological cardiac parameters such as heart rate (HR), heart period (HP), diastolic diameters (DD), systolic diameters (SD), diastolic intervals (DI), systolic intervals (SI), cardiac rhythmicity (arrhythmia index, AI) and cardiac performance (% fractional shortening, FS) will be determined for each fly group to detect cardiac defects using established protocols^13^. To avoid any circadian variability in cardiac function, all assays will be performed between ZT4 and ZT8 for both aging and DCM models. Briefly, semi-intact hearts are prepared in an artificial hemolymph as previously reported^13^. Direct immersion optics (Olympus Umplfln 10XW objective) connected with BX43F-1-3 microscope (Olympus Corp.) is used in conjunction with a digital high-speed camera at 200 frames/sec, (Hamamatsu Flash 4 camera) to record 30-s movies of beating hearts. Bright-field CXD movies were captured (400 × 300-pixel resolution) using HC Image (Hamamatsu Corp.). The pixel-to-micron ratio was calculated and CXD movies were converted to AVI movies for machine learning or SOHA analyses. Cardiac function was analyzed from the high-speed movies using the semi-automatic optical heartbeat analysis (SOHA) software as previously reported^13^ in addition to the deep learning methodology as described below.

### Dataset curation

A standard library of high-speed cardiac optical recordings is procured for training. We employ 54 training videos, with an 85%/15% train/validation split. Training videos are captured in grayscale format with a total of 500 frames per video from a complete 6000 frames, indicating 2.5 s of recording. Training videos include 47 wild-type videos and 7 videos with genetically engineered flies. Supplementary Table 1 depicts the group numbers and genotypes of the fly heart videos used for training. Researchers used the Computer Vision Annotation Tool (CVAT) software to produce high-quality masks on a per-frame basis. Annotations spanned identifiable heart walls—annotations were clipped in the presence of pericardial occlusion, change of heart regions, and unclear tissue. Annotation masks were reviewed for accuracy before usage in deep-learning experiments. Testing videos are captured in identical formats. We procure 177 videos for experimental testing, with $n=46$ for 1-week males, $n=43$ for 1-week female $n=44$ for 5-week males, and $n=44$ for 5-week females.

### Deep-learning development and training

The model design was performed in Python using the PyTorch deep learning framework^37^. Our study employs a modified Attention-UNet architecture for semantic segmentation^22^. The model contains a symmetric encoder/decoder architecture containing 8, 16, 32, 64, and 128 filters, employing a rectangular convolutional kernel. Validation loss was optimized via Dice Loss^42^. Our model employs a random weight initialization, the Adam^43^ optimizer and a 16-image batch size. For experimental testing, the model epoch with the lowest validation loss was employed. During the training process, the neural network samples frames by its corresponding diameter, as measured via its human-annotated mask. 75 frames per measured diameter are randomly sampled to be included in the training dataset – this is done to minimize class imbalance between diastolic and systolic frames in our segmentation task. After frame sampling, 3D samples are constructed by appending frames at $(t-4,t,t+4)$ indices, allowing us to encode temporal information in 3D convolutions. After sampling and augmentation, the model used a total of 114750 images for training with 19350 images used for validation. The model was trained for a total of 30 epochs, yielding a total of approximately 30 compute hours. Models were trained on a Tesla P100 GPU on the UAB Cheaha supercomputer.

### Calculation of cardiac parameters

Once a video has been selected for analysis, each frame $t$ is converted into a three-channel image with the $t-4$ frame and the $t+4$ frame. Images are then processed via a neural network. On a Tesla P100, we estimate this process to take approximately 103 seconds for a video with 5990 frames (58.16 FPS). From sigmoidal activations, the user determines an acceptable threshold and region of interest (ROI) through visual confirmation. Using the provided values, the average heart diameter for each frame is measured. Processing codes for the identification of diastolic intervals (DI) and systolic intervals (SI) are provided in the paper repository (GitHub). The diastolic diameter (DD) for each DI is taken to be the largest diameter attained during the DI. Similarly, the systolic diameter (SD) for each SI is taken to be the smallest diameter attained over its duration. We derive additional cardiac parameters from the collected DD, SD, DI, and SI statistics. Equations for fractional shortening (FS), ejection fraction (EF), heart period (HP), heart rate (HR), and arrhythmia index (AI) can be found in the literature^6^. Stroke volume and cardiac output calculations follow those in Klassen et al.^16^, along with peak contraction velocity and peak contraction to relaxation latencies^16^. Velocity information is procured via numeric differentiation of frame-level time-resolved diameter data. Extraction of beat-level data enables the capturing of SI and DI arrhythmias, referred to as tachycardiac and brachy-cardiac arrythmia. After identifying all SIs and DIs at the beat level, tachycardiac data is selectively filtered from all SI events with a length of over 0.5 s. Brachy-cardiac events are extracted from DI data with a length of over 1.0 s. These filtering parameters are extracted from analysis performed by Occur et al.^4^.

### Experimental validation

We procure high-speed videos of $n=46$ for 1-week males, $n=43$ for 1-week females, $n=44$ for 5-week males, and $n=44$ for 5-week females to experimentally test our model. For each heart, we calculate time-resolved beating patterns and cardiac statistics using our deep-learning approach. For each heart, an end-to-end analysis takes approximately 2 min. Next, each heart was identically examined in the canonical software for small-animal cardiac analysis, titled SOHA^13^. We employ the SOHA software for experimental validation of our model using blind data. Statistics comparing age-dependent phenotypes are calculated using generally available Python packages. For comparison of aging groups, we calculate T-Test significance values. Furthermore, for a quantitative view of our model performance, we calculate the pairwise coefficient of determination $({R}^{2})$ score via the Scikit-Learn Python library^44^. A close agreement between deep-learning calculated data and SOHA data indicates high model performance.

### Classification of *Drosophila* age

Calculated cardiac statistics are exported from experimental studies. A dataset is labeled with cardiac statistics and corresponding fly ages (1 week, 5 weeks). We produce a predictive model combining calculated cardiac statistics and fly age via logistic classification. To fit this logistic model, we employed the Scikit-Learn Python library. Our logistic model was fit on experimentally predicted DD, SD, FS, DI, SI, HP, and AI parameters. A *k*-fold cross-validation with $k=5$ folds was used to evaluate model performance. Such a configuration achieved a high testing accuracy with our model, as quantified in the Results section. To elucidate the connection between cardiac physiological parameters and age dependence, we employ the SHapley Additive exPlanations (SHAP)^45^ technique. Doing so enables model transparency and insights into the main determinants of aging phenotypes.

We also investigate the use of deep learning models for the classification of *Drosophila* age from cardiac recordings. For this task, the sum of the squared difference of each pixel between a frame ${t}_{0}$ and frame t is saved as time series data and is normalized between 0 and 1. This data is binarized with a threshold of 0.5. The resulting time series is composed of alternating regions of consecutive ones and consecutive zeros. The frame corresponding to the center of each region was saved until 96 frames had been collected. This along with the duration between each frame was taken as the input for the model. The model passes the 96-frame clip through 3 convolution blocks each consisting of two 2D convolutional layers followed by a max pooling layer. The result is then flattened and combined with the duration data before being passed into three dense layers and a final sigmoid output layer. Our dataset consisted of $n=118$ for 1-week males, $n=156$ for 1-week females, $n=121$ for 5-week males, and $n=98$ for 5-week females. Again, we utilized k-fold cross-validation to evaluate model performance with $k=5$ folds.

### Statistics and reproducibility

All statistical analyses were performed with GraphPad Prism 9. Significance in cardiac function was determined using one-way ANOVA with two-sided unpaired T-test as previously described^6,10–12^_._ To rule out sex-specific differences, our analysis will be carried out in both male and female flies. In addition to sex, the genetic background of the flies, temperature, and light/dark cycle are important biological variables that were used. N numbers for each assay are shown in figure legend from a minimum of 3 repeated experiments, as previously described^6,33^.

### Reporting summary

Further information on research design is available in the Nature Portfolio Reporting Summary linked to this article.

### Supplementary information

Supplementary information

Description of Additional Supplementary Materials

Supplementary Video

Supplementary Data 1

Reporting Summary

## Data Availability

All the data used within this paper and the connected findings of this study are available as Supplementary Data 1.

## Code availability

The copyright codebase as well as example notebooks for analysis are being prepared and will be made publicly available on GitHub (GitHub - ymelkani/Drosophila-Cardiac-Analysis) under a GPL2.0 license upon acceptance.
