# Supplementary material for: Automated assessment of cardiac dynamics in aging and dilated cardiomyopathy Drosophila models using machine learning
Source: Commun Biol. 2024 Jun 7;7:702. doi: 10.1038/s42003-024-06371-7 (PMC11161577; doi:10.1038/s42003-024-06371-7)
Supplement: Supplementary file 1 — Supplementary information [file 42003_2024_6371_MOESM1_ESM.pdf]

Supplementary Materials for

Automated assessment of cardiac dynamics in aging and dilated cardiomyopathy Drosophila models using machine learning

Melkani et al., Correspondence and requests for materials should be addressed to G.C.M
(Email: girishmelkani@uabmc.edu)

This PDF file includes Supplementary Figures 1 to 8 with legends and Supplementary Table 1.

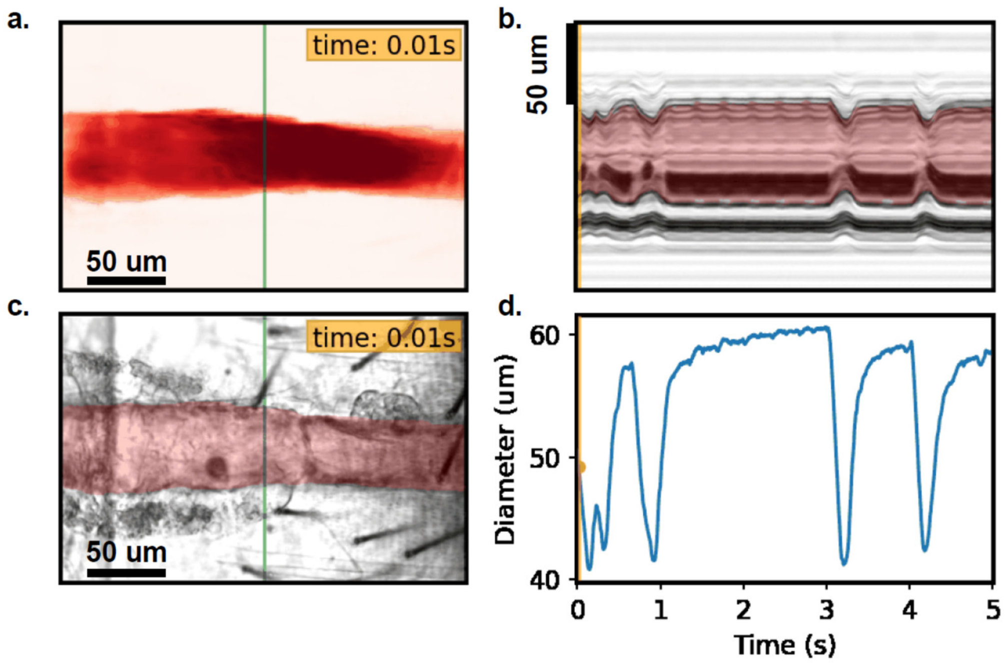

Supplementary Figure 1: Representative image of the output of the neural network. a. The per-frame sigmoid output of the neural network. **b.** Mechanical mode (M-Mode) image generated with overlaid neural network output. **c.** Raw video frame output with annotated heart-wall morphology (red). **d.** Time-resolved beating pattern calculated via c.

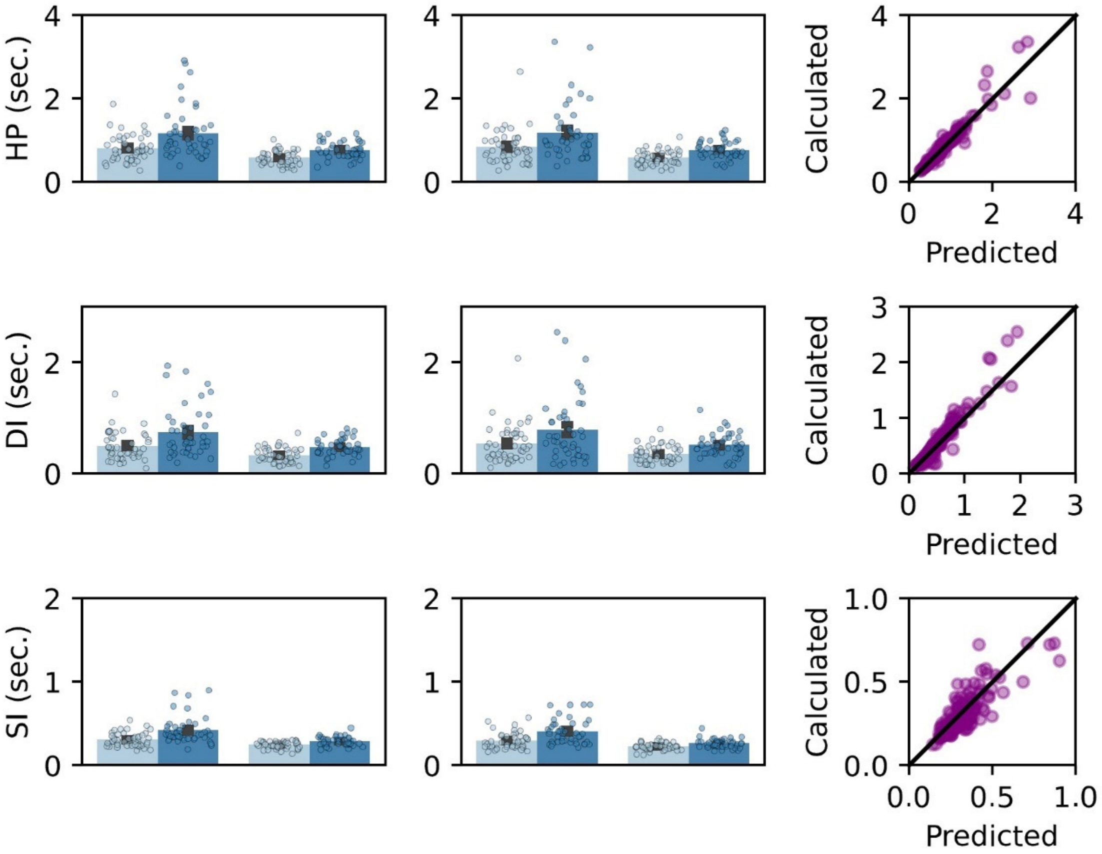

Supplementary Figure 2: Additional cardiac parameters calculated by neural network. (a) Heart period, in seconds, calculated by our model (left), SOHA (right), and agreement between two datasets (right). **(b)** Diastolic interval, in seconds, calculated by our model (left), SOHA (right), and agreement between two datasets (right). **(c)** Systolic interval in seconds, calculated by our model (left), SOHA (right), and agreement between two datasets (right). Statistics are calculated via the use of a restricted ROI, selected by a trained user. Age-dependent statistics compared with one-way ANOVA with two-sided unpaired t-test.

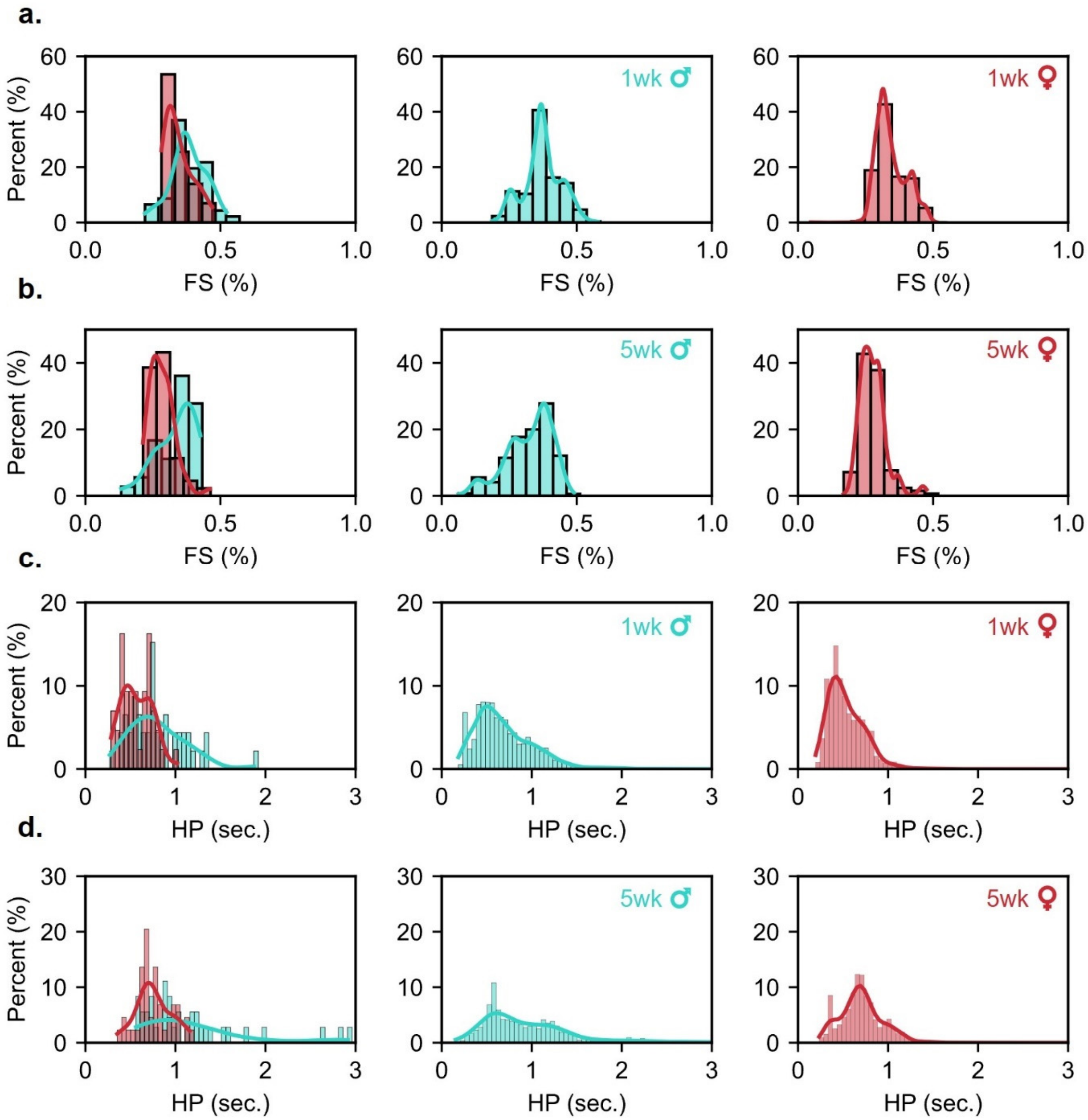

Supplementary Figure 3: Beat-level parameter distributions. Selected parameters (FS, HP) are visualized on a per-beat, per-heart basis along with corresponding cohort-level data. **(a)** Fractional shortening of cohort (left), 1wm (middle) and 1wf (right) hearts. **(b)** Fractional shortening of cohort (left), 5wm (middle) and 5wf (right) hearts. **(c)** Heart period of cohort (left), 1wm (middle) and 1wf (right) hearts. **(d)** Heart period of cohort (left), 5wm (middle) and 5wf (right) hearts. Aging flattens and shifts the FS distribution downwards (less cardiac function), and HP distribution upwards (longer).

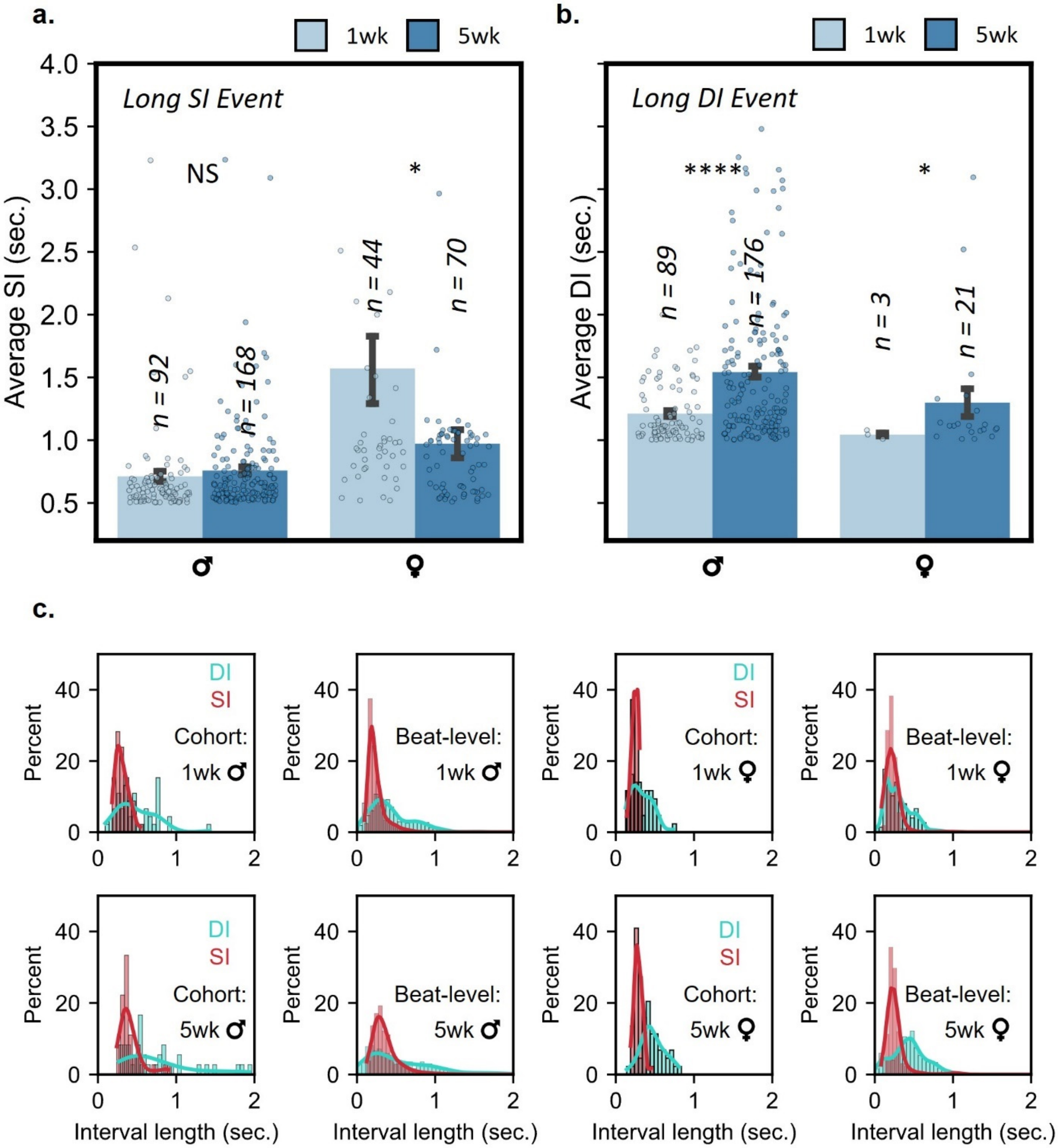

Supplementary Figure 4: Model detected brachy and tachycardiac arrhythmias. Brachy and tachycardiac arrhythmias are selected by filtering the beat-level SI with length above 0.5s and DI above 1.0s. (A, B) Model-detected brachy and tachycardiac arrhythmias are selected and analyzed. T-test analysis suggests significant differences in arrhythmia length with aging by analyzing beat-level data. (C) Cohort-level and beat-level distributions of SI and DI model-detected data. Age-dependent statistics compared with one-way ANOVA with two-sided unpaired t-test.

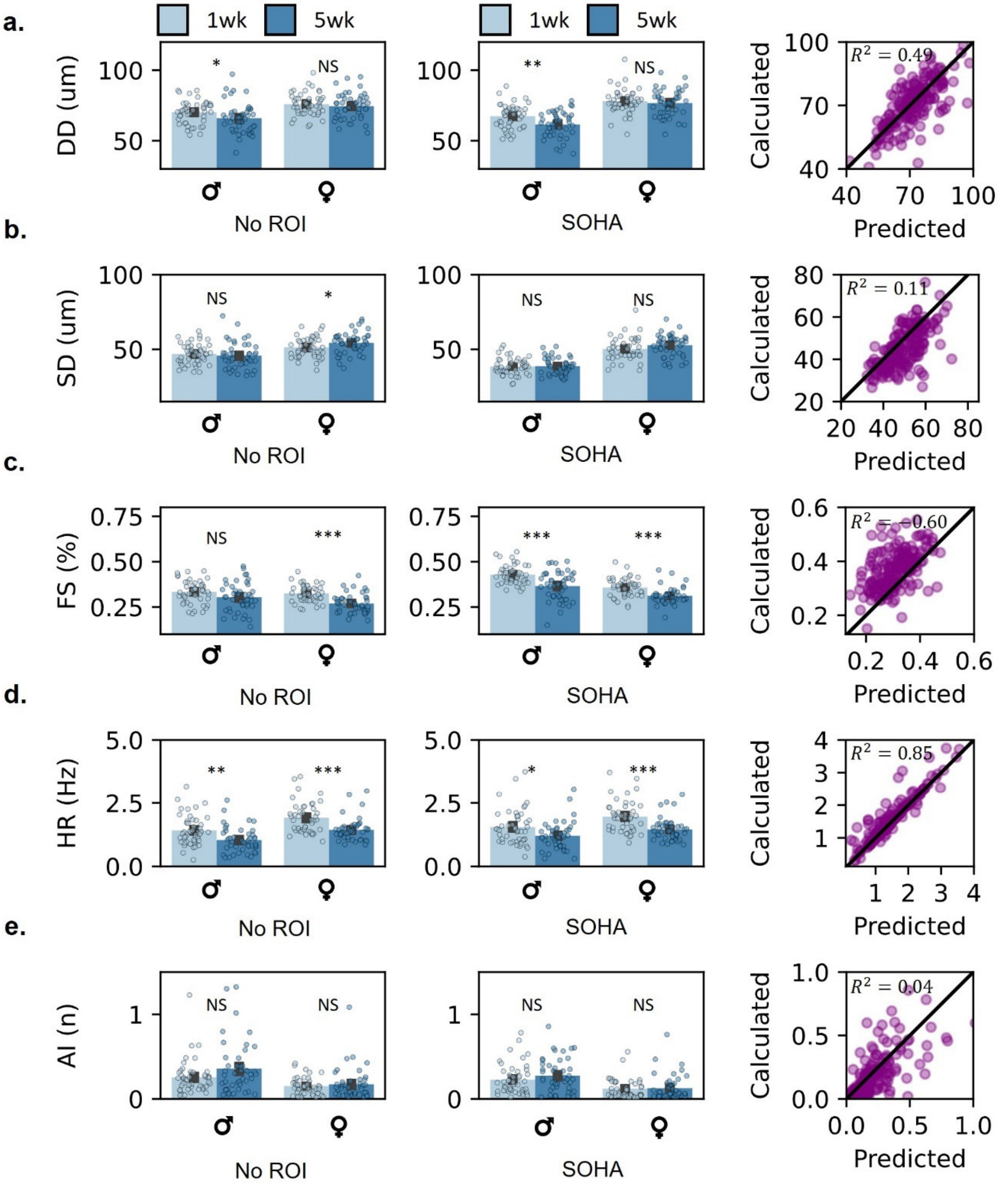

Supplementary Figure 5: Cardiac parameters calculated by the neural network without provided ROI or threshold. (a) Diastolic diameter, in microns, is calculated by our model (left), SOHA (right), and agreement between two datasets (right). (b) Systolic diameter, in microns, calculated by our model (left), SOHA (right), and agreement between two datasets (right). (c) Radial contractility (fractional shortening), in percentage, calculated by our model (left), SOHA (right), and agreement between two datasets (right). (d) Heart rate, in Hertz, is calculated by our model (left), SOHA (right), and agreement between two datasets (right). (e) Beating dysrhythmia (arrhythmia index) calculated by our model (left), SOHA (right), and agreement between two datasets (right). All error

bars report ± SEM. Age-dependent statistics compared with one-way ANOVA with two-sided unpaired t-test. Statistics are calculated without the use of a restricted ROI.

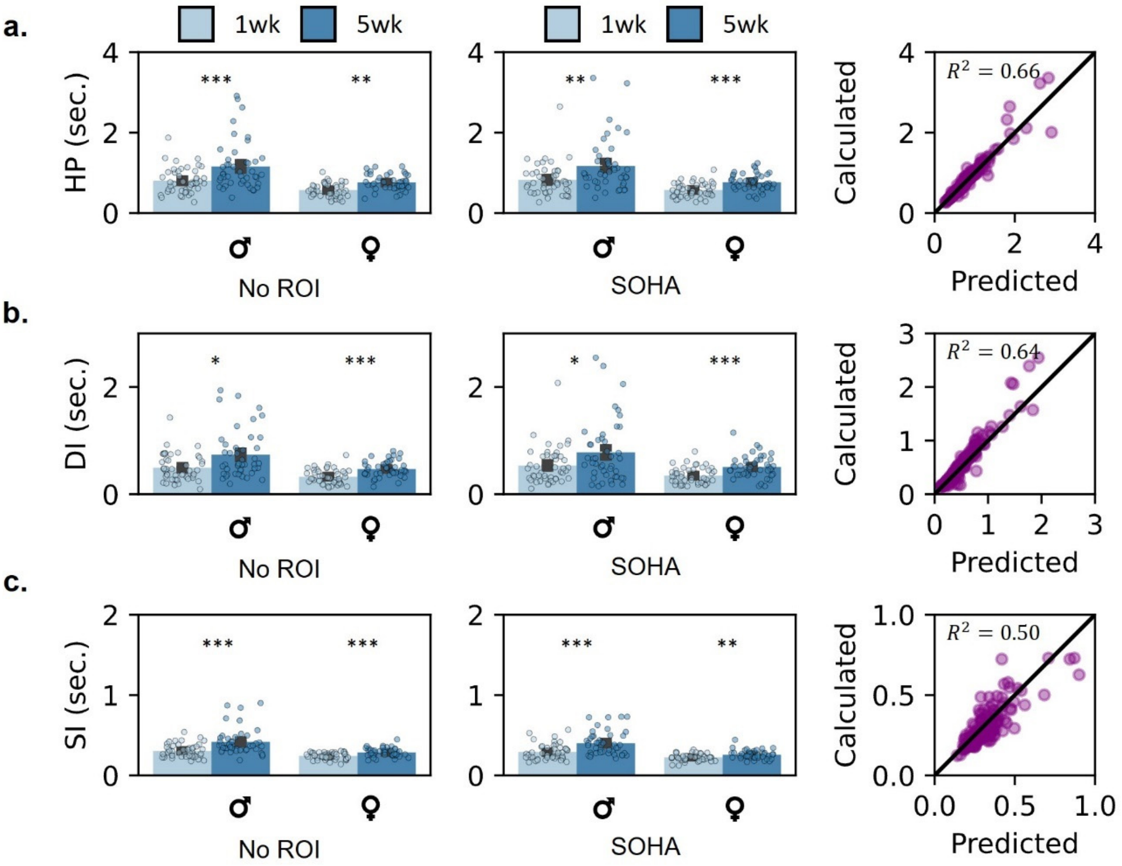

Supplementary Figure 6: Additional cardiac parameters calculated by neural network without provided ROI or threshold. (a) Heart period, in seconds, calculated by our model (left), SOHA (right), and agreement between two datasets (right). (b) Diastolic interval, in seconds, calculated by our model (left), SOHA (right), and agreement between two datasets (right). (c) Systolic interval in seconds, calculated by our model (left), SOHA (right), and agreement between two datasets (right). Age-dependent statistics compared with one-way ANOVA with two-sided unpaired t-test. Statistics are calculated without use of a restricted ROI.

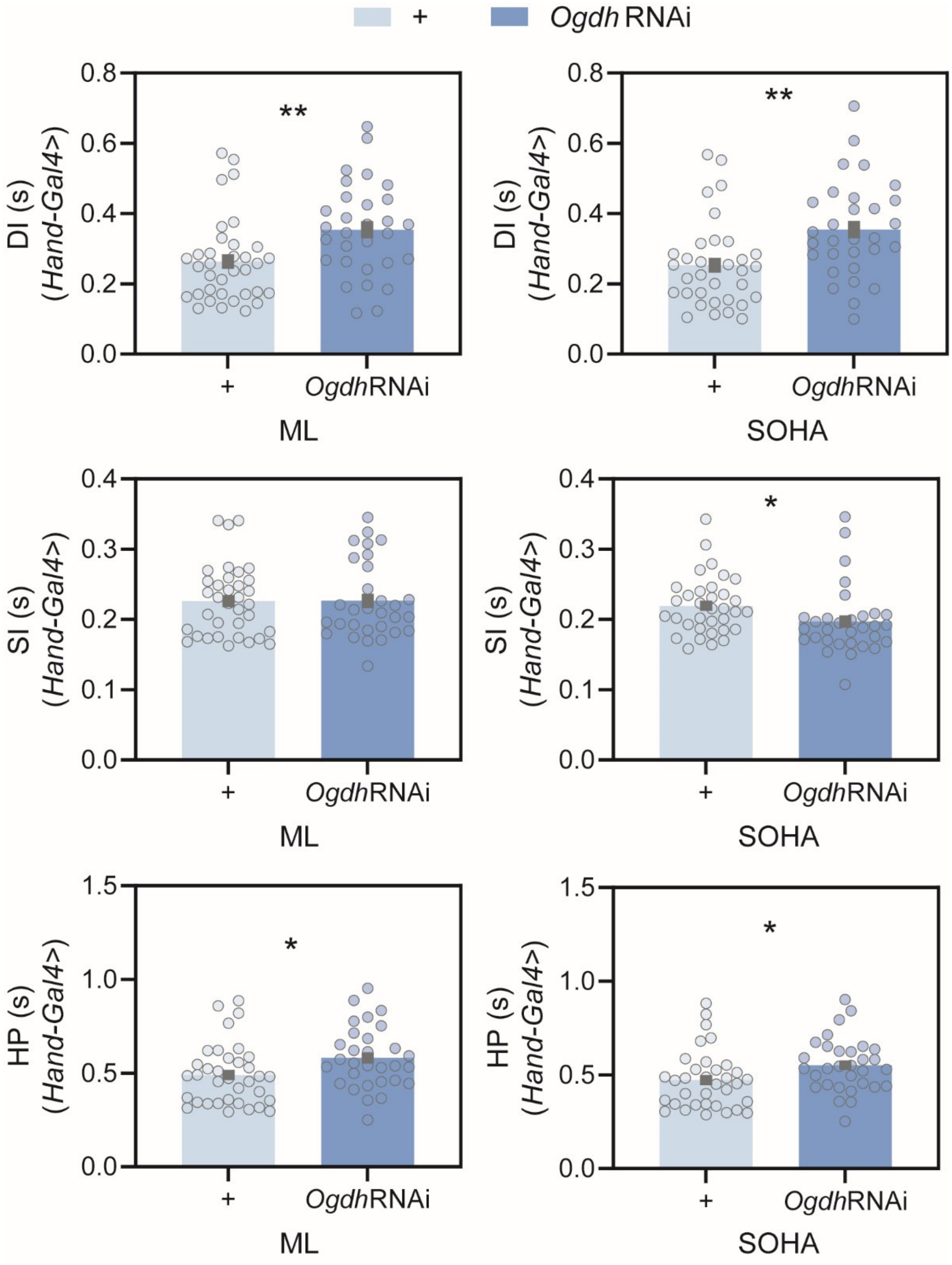

Supplementary Figure 7: Cardiac-specific knock-down of *Odgh* leads to cardiac dysfunction. Histograms of cardiac physiological parameters 3-weeks control (*Hand/+)* and *Hand>Ogdh RNAi*

has compromised (a) diastolic intervals (DI), (b) systolic intervals (SI), and (c) heart period, analyzed using machine learning (ML) left, and SOHA right panels. N = 35 (*Hand/+)* and 30 *Hand>Ogdh RNAi for* ML; and N=35, (*Hand/+)* and 31 *Hand>Ogdh RNAi* for SOHA were represented as mean ± SEM. Knock-down of *Ogdh* with age-matched control statistics compared with one-way ANOVA with two-sided unpaired t-test.

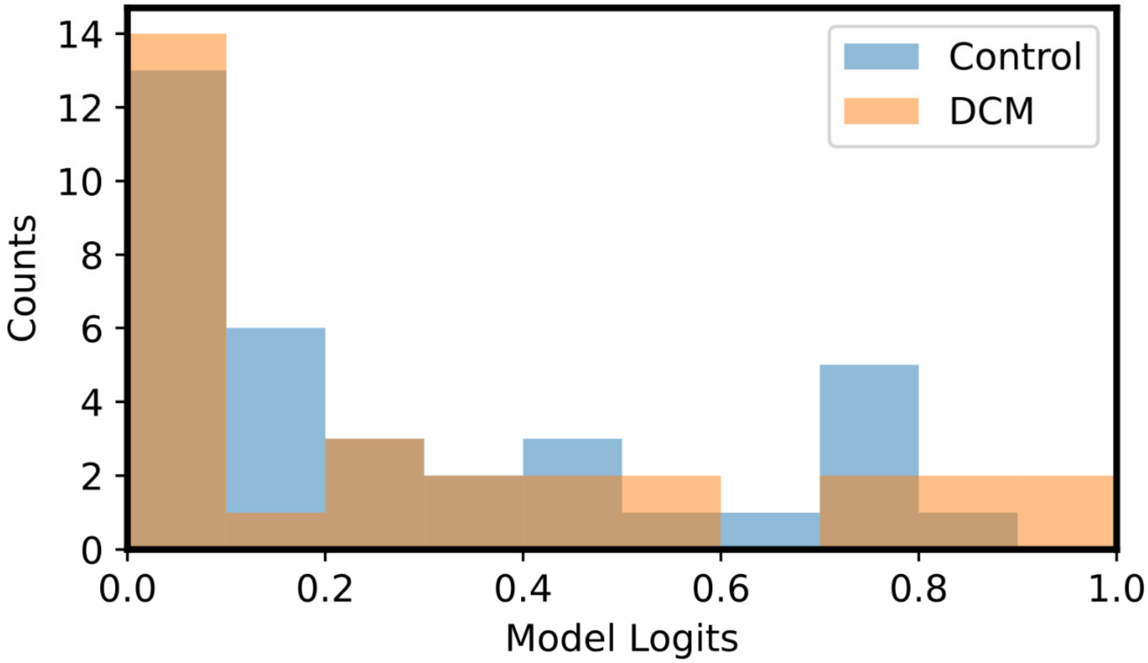

Supplementary Figure 8: Aging model predictions for wildtype (n=35) and DCM (n=30) hearts. Distribution of average model predictions from k=5 folds. Predictions toward one indicate that the sample mimicked an aged heart, while predictions toward zero indicate that the sample mimicked a young heart

Supplementary Table 1: Summary of heart videos for train including age, sex, and genotype

Genotypes	Age and sex of the each genotypes							**Total**
	1wm	1wf	3wm	3wf	4wf	5wm	5wf	
CS (wildtype)	6	9	1	5	2	9	13	**45**
W^{1118} (wildtype)	2							**2**
Ogdh knock-down				5	2			**7**
**Total**	**8**	**9**	**1**	**10**	**4**	**9**	**13**	**54**

w=week
m=male
f=female
