# Supplementary material for: Automated assessment of cardiac dynamics in aging and dilated cardiomyopathy Drosophila models using machine learning
Source: Commun Biol. 2024 Jun 7;7:702. doi: 10.1038/s42003-024-06371-7 (PMC11161577; doi:10.1038/s42003-024-06371-7)
Supplement: Supplementary file 2 — Description of Additional Supplementary Materials [file 42003_2024_6371_MOESM2_ESM.docx]

**Description of Additional Supplementary Files**

**File name:** Supplementary Data 1

**Description:** All the source data underlying graphs and charts

**File name:** Supplementary Video 1

**Description:** Animated Supplementary Video S1 shows the output of the neural network as shown step-by-step in Supplementary Figure 1
