# Supplementary figures and images for: Automated assessment of cardiac dynamics in aging and dilated cardiomyopathy Drosophila models using machine learning
Source: Commun Biol. 2024 Jun 7;7:702. doi: 10.1038/s42003-024-06371-7 (PMC11161577; doi:10.1038/s42003-024-06371-7)

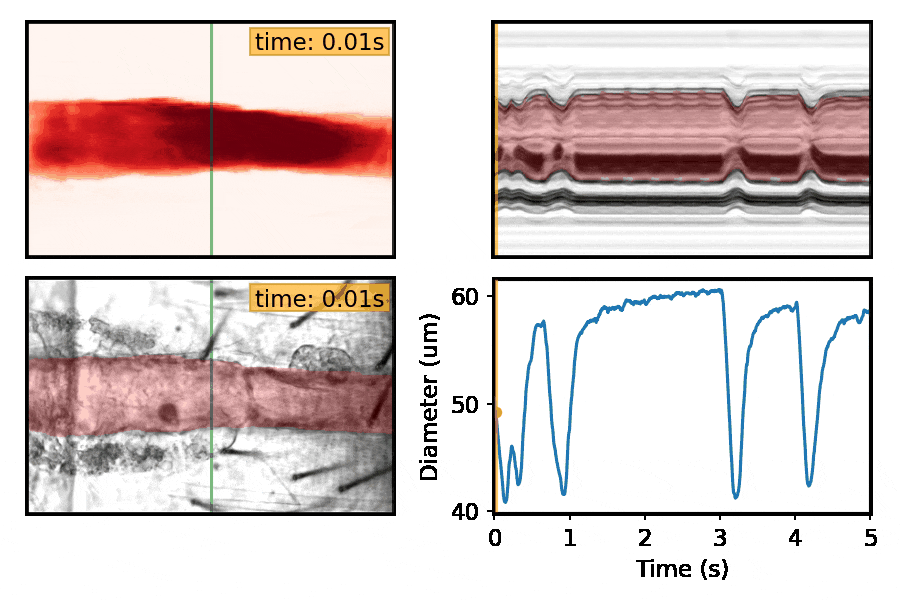

Supplement: Supplementary file 3 — Supplementary Video [file 42003_2024_6371_MOESM3_ESM.gif]
